# Adaptive Simplex Architecture for Safe, Real-Time Robot Path Planning

**DOI:** 10.3390/s21082589

**Published:** 2021-04-07

**Authors:** Tudor B. Ionescu

**Affiliations:** Human-Machine Interaction Group, Vienna University of Technology, 1040 Vienna, Austria; tudor.ionescu@tuwien.ac.at

**Keywords:** simplex architecture, path planning, machine learning, real time, obstacle avoidance

## Abstract

The paper addresses the problem of using machine learning in practical robot applications, like dynamic path planning with obstacle avoidance, so as to achieve the performance level of machine learning model scorers in terms of speed and reliability, and the safety and accuracy level of possibly slower, exact algorithmic solutions to the same problems. To this end, the existing simplex architecture for safety assurance in critical systems is extended by an adaptation mechanism, in which one of the redundant controllers (called a high-performance controller) is represented by a trained machine learning model. This model is retrained using field data to reduce its failure rate and redeployed continuously. The proposed adaptive simplex architecture (ASA) is evaluated on the basis of a robot path planning application with dynamic obstacle avoidance in the context of two human-robot collaboration scenarios in manufacturing. The evaluation results indicate that ASA enables a response by the robot in real time when it encounters an obstacle. The solution predicted by the model is economic in terms of path length and smoother than analogous algorithmic solutions. ASA ensures safety by providing an acceptance test, which checks whether the predicted path crosses the obstacle; in which case a suboptimal, yet safe, solution is used.

## 1. Introduction

Robot applications involve motion planning problems, for many of which there exist machine learning (ML) model-based approaches (e.g., collision-free path and trajectory planning, assembly, bin picking and placing, etc.). Although ML is typically used when a computational algorithm does not exist or would be impractical (e.g., due to complexity of error proneness), ML-based solutions to problems that allow computational algorithms provide advantages in terms of performance thanks to the time-efficiency of machine learning model scorers (i.e., algorithms that process trained ML models to produce predictions for given inputs).

*Motivation.* Collaborative robot applications can especially benefit from employing fast ML models for motion planning with dynamic collision avoidance (e.g., with body parts and other moving objects). Yet, safety concerns currently prevent the use of ML-based motion planning models in practical collaborative robot applications [1]. As opposed to deterministic computational algorithms, ML models cannot ensure solution correctness in all conceivable situations, especially for cases that are outside the scope of the training data [1,2]. To illustrate this, we consider the motivating example of dynamic obstacle avoidance, which requires (re)planning robot motion paths in a potentially unbounded search space to avoid collisions with (moving) obstacles. Depending on the shape and dynamics of the obstacles, optimal computational solutions to this problem may perform poorly in terms of execution time, whereas ML models cannot ensure correctness and optimality for every possible input case.

**Figure 1:**
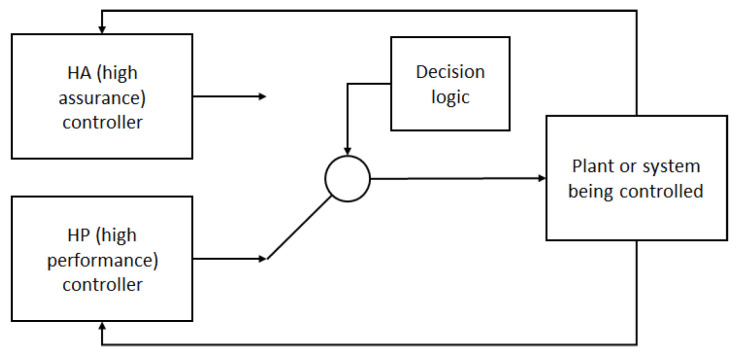
The original simplex architecture (SA) [3].

*Problem.* Against this background, this paper addresses the question of how to use ML solutions in practical robot applications so as to achieve the performance level of the ML solution in terms of speed and reliability, and the safety level and accuracy of the algorithmic solution at all times. This basic formulation of the problem is similar to that of the simplex architecture (SA) [3] illustrated in Figure 1. In the original SA, a system (i.e., plant) can be controlled by a high-assurance (HA) and a high-performance (HP) controller or control subsystem. The HP has a higher performance at the cost of higher complexity and, thus, lower reliability. The HA is a simpler and more reliable control subsystem, which is certified together with the decision logic. The SA initially uses the HP and checks if its output is safe by asserting that it is within a so-called recovery region, in which the system operates safely. If that is not the case, the decision logic permanently switches to the output of the HA. The HP is reactivated when the system is restarted or after a service upgrade. The main advantage of using the SA in safety critical systems is that one only needs to certify the HA and the decision logic, while being able to use an optimal, yet less reliable, HP most of the time during operation. The key element of the SA is the decision logic used to determine whether the HP’s output is within the recovery region.

*Contribution.* The original simplex architecture is extended by a dynamic adaptation mechanism, which allows it to leverage the potential of ML models without compromising safety. The proposed adaptive simple architecture (ASA) improves on the original SA architecture by (i) extracting an acceptance test (AT) from the decision logic, which tests the output of the HP and passes the test verdict to the decision logic, (ii) using a trained ML model as the HP control subsystem or program, (iii) using operational data to continuously improve the ML model during operation, (iv) updating the ML model used as the HP continuously, and (v) dynamically switching between the HP and SA at runtime, depending on the AT verdict. To evaluate the ASA, a neural network model for path planning is used as the HP planner and a simple, reliable, and fast path planning algorithm as the HA planner. The ML model is trained on a machine learning platform using an optimal, yet slow, path planning algorithm. In operation, the ML model is improved by adding the input cases for which it fails to the training set. The HP is updated at the runtime whenever an improved ML model becomes available. The ASA thus ensures that the path planning module performs (1) as reliably as the computational path planning algorithm used as the HA, (2) as fast as the ML model scorer used as part of the HP, and (3) nearly as optimally in terms of accuracy as the optimal path planning algorithm used to train the ML model. Thanks to the second property, the ASA is suitable for motion planning scenarios in which frequent replanning is required (e.g., for dynamic obstacle avoidance).

*Evaluation.* The ASA is evaluated on the basis of a dynamic obstacle avoidance application for a generic six degrees of freedom (6-DOF) manipulator and a moving obstacle. To this end, a smooth path planning algorithm based on minimizing the length of a Lamé curve (i.e., super ellipse) around an obstacle is introduced and used to train a ML model, which is used as the HP planner in the ASA. The HA planner is represented by a fast and safe heuristic used to optimize the Lamé curve parameters. The proposed ASA and dynamic obstacle avoidance algorithm are implemented on top of an open source, web-based 6-DOF robot simulator [4], which is available online [5]. As part of the evaluation, two human–robot collaboration (HRC) scenarios from the manufacturing domain are considered as potential use cases of the proposed ASA. In these scenarios, the emphasis is put on increasing the flexibility of HRC in manufacturing without compromising safety. The performances of the proposed path planning method are compared to those of four other state-of-the-art methods. The proposed approach is validated experimentally using a Universal Robot 5 6-DOF robot in the Industry 4.0 Pilot Factory of the Vienna University of Technology, Vienna, Austria.

The paper is structured as follows: Section 2 discusses different approaches that are related to the methods introduced in this paper. Section 3 introduces the adaptive simplex architecture in more detail. Section 4 introduces an application of the proposed ASA to path planning with dynamic obstacle avoidance for a 6-DOF robot. Section 5 presents an evaluation of the approach. Section 6 discusses the results obtained during the evaluation, as well as the limitations of the proposed approach. Section 7 concludes the paper by highlighting the novel and useful aspects of the proposed approach.

## 2. Related Work

### 2.1. Simplex Architectures in Robotics

Different versions of the SA have been used in robotic applications for autonomous vehicle [6,7,8] and drone flight path planning [9]. Where, in reference [9], the original SA is used, the other approaches use ML models as the HP planners. In references [6] and [7], convolutional neural networks are trained using images of a vehicle circuit track and deployed as the HP planner. The HP planner, however, is not updated at the runtime. In reference [9], a motion planning algorithm from the Open Motion Planning Library is used as the HP planner for a surveillance drone. In references [8] and [9], reinforcement learning-based HP planners are used for autonomous vehicle path planning. In reference [8], the so-called neural simplex architecture (NSA) is introduced, which is similar to the proposed ASA. The NSA uses a deep neural network and reinforcement learning with rewards and penalties to train a ML model that is used as the HP planner in a rover navigation application with static obstacle avoidance. The NSA switches from HP to SA upon encountering an unrecoverable HP output, during which the ML model is retrained using an initial training set to which the data collected during the operation are added. The additional data contain both successful and unsuccessful cases. After retraining, a new ML model is deployed as the HP planner, and the control is switched back from the HA to HP.

ASA differs from NSA [8] in several significant ways. ASA uses a deterministic path planning algorithm (rather than a stochastic random walk heuristic) to generate training data for the supervised training of a feedforward neural network (or any other suitable ML method). This leads to simpler models, which require fewer training samples (i.e., ~10,000 samples in the case of ASA rather than ~one million samples in the case of NSA). In ASA, the ML model is retrained only using the cases that fail the acceptance test, thus further reducing the size of the training set. Regardless of the performance of the HP planner and the state of retraining, the ASA continuously switches back and forth between the HP and HA, because each output of the HP is subjected to a fast AT. Hence, the performance of the ASA in terms of speed is maximized, provided that ML model scoring is faster than the HA (which is the case in curve-based path planning).

In reference [1], the authors note that deep learning methods can produce unsafe predictions for inputs that were not covered by the training process. To tackle this issue, these authors proposed an autoencoder-based anomaly detection method that supervises the input and output of the model and reverts to a safe prior behavior (e.g., limiting the speed such that the robot can stop within the known free space) when a new, potentially hazardous situation is detected. Reference [1] differs from the proposed ASA approach in that it uses machine learning models both for high-performance, self-improving navigation and for switching to a safe behavior. By contrast, ASA uses a reliable deterministic (rather than probabilistic) acceptance test and a simple and reliable algorithmic alternative for the high-assurance controller (i.e., path planner). This allows the ASA to use machine learning in safety critical applications, which are subjected to safety certifications.

### 2.2. Machine Learning Approaches to Motion Planning with Obstacle Avoidance

Machine learning approaches have been proposed for mobile robot navigation with collision avoidance. Convolutional neural networks (CNN) [10] and reinforcement learning [11] are used for indoor obstacle avoidance. The CNN takes, as input, raw images and produces control commands, whereas, with deep reinforcement learning, there is no need for manually designed features and prior demonstrations to train the model. In reference [12], training data are generated using a simulation environment that takes into consideration physical interactions to implement deep reinforcement learning for mobile robot navigation. Reinforcement learning is also used in references [13] and [14] for navigation in complex dynamic environments.

In the case of robot arms (i.e., serial manipulators), the current approaches to obstacle avoidance use various combinatorial search strategies to find suitable robot poses along a trajectory, e.g., evolutionary algorithms [15,16], different versions of the Rapidly Exploring Random Tree method [17,18,19,20], etc. The common aspects in these approaches are that they take into consideration the potential collisions of all the joints of the robot with the obstacle(s), and, in so doing, they require repeated computations of inverse kinematics for a high number of robot poses resulting from the different combinatorial search strategies being used. As a result, these approaches have high computational demands, which leads to slow robot reactions to moving obstacles. To accelerate the trajectory planning for serial manipulators in the presence of (dynamic) obstacles, there exist approaches that leverage different machine learning techniques. In reference [21], the authors propose a reinforcement learning-based strategy for 6-DOF manipulators, which starts with planning an obstacle avoidance path for the terminal element of the manipulator (e.g., tool center point (TCP)). Subsequently, different robot poses are tested along this path so as to avoid collisions between any of the robot joints and the obstacles. In references [22,23], a method for printing onto unknown and arbitrarily shaped 3D substrates using a 6-DOF manipulator is introduced. Part of this challenge consists in planning a toolpath that follows the surface of the substrate, which entails avoiding collisions with the surface while maintaining a constant distance from it. This is useful, e.g., for landscape architecture models. To this end, a Universal Robot 10 arm endowed with an “eye-in-hand” RealSense D435 is used. To support the 3D printing process in real time, a neural network is trained, which relates the position and size of a generic obstacle to a set of poses the robot needs to take in order to avoid collision with that given obstacle [23]. In reference [24], a Q-learning-based approach (i.e., a kind of reinforcement learning) is proposed for solving the robot arm path planning problem while accounting for joint motions, whereby approximate regions are used to define the new state space and joint actions instead of accurate measurements. In reference [25], an approach based on regenerative recurrent neural networks is proposed for solving the collision-free maneuvering problem for generic many-joint arms of up to 80 joints. The so-called Soft Actor-Critic with Hindsight Experience Replay (SAC–HER) method [26] uses reinforcement learning to plan trajectories for a dual 3-DOF arm robot. The planning occurs jointly for both robots, as if they were part of the same entity. As a result, the planning is performed for 6-DOF. A similar Soft Actor-Critic (SAC)-based method [27] extends the SAC–HER approach by using a dual 7-DOF arm robot. Due to the complexity of the problem, this method does not always succeed in producing a viable path due to self-collisions and other issues.

The approach proposed in this paper to dynamic obstacle avoidance uses a simple machine learning approach based on the multilayer perceptron (a basic type of artificial neural network) technique to estimate, in real time, the parameters of a Lamé curve representing an obstacle avoiding toolpath between origin and target. The proposed method does not take the joint positions of the 6-DOF robot into account. Instead, it introduces the following constraints: (i) the orientation of the tool is fixed during the movement so as to eliminate two degrees of freedom, (ii) the path is planned in 2D space in a projection plane so that the robot can either avoid obstacles in the horizontal (i.e., ground) or a vertical plane (i.e., orthogonal to the ground) that contains the origin and target points, and (iii) the access to the robot is physically restricted to a safe collaboration zone.

### 2.3. Iterative Learning and Model Predictive Control-Based Method

In reference [28], the problem of path tracking of industrial robots is addressed. The algorithm corrects a preplanned path through a new iterative learning control (ILC) method called the calibration based ILC, which identifies the kinematic parameters along the path in a workspace. In reference [29], a trajectory planning method, which tracks the movement of a human operator, is proposed. Through initial learning by demonstration, the behavior of the robot evolves into a cooperative task, where the human coworker is allowed to modify the motion trajectory and speed. As a result, bimanual human–robot collaboration applications can be implemented. In reference [30], a robust cascade path-tracking control method is proposed, which achieves good positional control performances for 6-DOF industrial robots. The method seeks an optimal solution for tracking the desired position profiles accurately and robustly. The model predictive control-based algorithm introduced in reference [31] uses the distance between the end effector and one or several moving obstacles to plan a feasible trajectory for a 6-DOF robot. Intuitively, this approach comes close to the basic idea behind the path planning method proposed in this paper. However, in reference [31], closed-loop control rather than machine learning is used.

### 2.4. Analytic, Curve-Based Path Planning Methods

Analytic, curve-based path planning methods [32] represent an alternative to the heuristic path planning methods discussed before. The solution entails finding a curve equation that, when plotted, avoids any obstacles between the origin and target locations. In reference [33], a method is proposed that leverages support vector machines (SVM) to generate a smooth collision-free path between two points in 2D or 3D space. The SVM is used to classify the obstacles’ points in two clusters. The results are then used to fit a curve (i.e., a smooth path) between the points from the two classes (i.e., the two clusters). The average planning time achieved by this method for one obstacle is 175.8 ms (for the scoring the SVM) and 3.3 s for generating a path on an unspecified computer from the early 2000s. Other approaches use parametric curves, such as Bezier [34,35] or Clothoid [36] curves, to generate collision-free paths for mobile robots and vehicles. These parametric curves are flexible and can be used to generate collision-free paths in complex environments. In this paper, the Lamé curve, which has a simpler expression than the Bezier and Clothoid curves, is used. In addition, the Lamé curve has a fixed set of parameters that can be optimized for a given obstacle shape. This allows to train a ML model able to estimate these parameters more easily. In robotics, the Lamé curve has been used in smooth trajectory planning for parallel [37] and delta [38,39,40] robots. In this paper, we apply the Lamé curve to compute smooth collision-free toolpaths for an industrial robot arm.

## 3. The Adaptive Simplex Architecture

**Figure 2:**
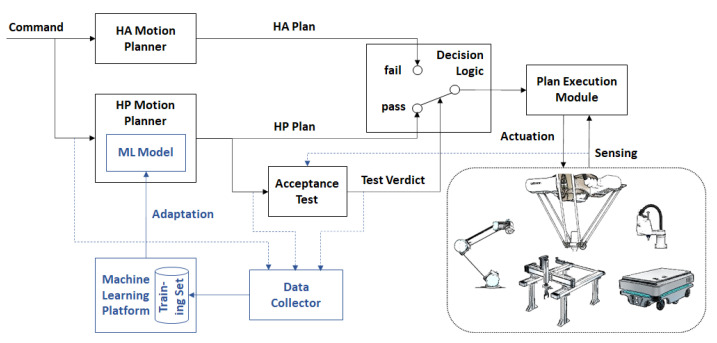
The proposed adaptive simplex architecture (ASA). The elements in blue illustrate the extensions to the original simplex architecture (SA). HA: high-assurance, HP: high-performance, and ML: machine learning.

This section describes the extensions to the original AS architecture shown in Figure 1 that allow the use of a ML model for motion planning as the HP planner and the continuous adaptation of that model during runtime. The proposed adaptive simplex architecture (ASA) is shown in Figure 2. The ASA is implemented as a software module, which is invoked using a command to generate a collision-free motion plan, be it a path or trajectory. ASA uses an acceptance test (AT) as the decision logic, which uses sensor data to assert that the motion plan fulfills all the requirements of the application, notably in terms of safety and reliability (e.g., a collision-free path or trajectory). If the HP plan passes the acceptance test, it is forwarded to a Plan Execution Module. Otherwise, the HA plan generated by a simple, suboptimal, yet robust motion planner is forwarded to the execution module. The inputs for which the HP planner fails the AT are added by the Data Collector to the ML model training set managed by a Machine Learning Platform (i.e., a computational environment that is external to the robot’s controller). As a result, the training set is extended by new input cases. When a sufficient number of new input cases that fail the AT are collected, an improved ML model is trained using the extended training set. When an improved ML model becomes available, it replaces the current ML model used by the HP Planner during runtime.

In the following, the key aspects and components of the proposed ASA are described in more detail.

### Model Training and Runtime Adaptation

The original SA switches control from the HP controller to the HA controller permanently upon the first violation of the recovery region in order to ensure the safety of a critical system (e.g., an airplane). By contrast, the ASA emphasizes performance while maintaining safety. Therefore, it switches dynamically back and forth between the HP and HA planner. This dynamic behavior is facilitated by a reliable acceptance test (AT), which is capable of checking whether the HP planner’s output is correct and, therefore, safe. The purpose of emulating motion planning algorithms using ML models is to speed up the generation of motion plans during runtime thanks to the low algorithmic complexity of model scoring compared to that of heuristic, graph-based motion planning. Since a ML model may provide correct predictions for some inputs and incorrect predictions for others, the goal is to maximize the use of the faster HP planner while maintaining safety. An optimal, yet possibly slow computational motion planning algorithm can be used to train a ML model that is used as the HP planner, whereas a simple, fast, and reliable motion-planning algorithm can be used as the HA planner. This latter algorithm can be a faster, less accurate version of the same algorithm that is used to train the ML model.

**Figure 3:**
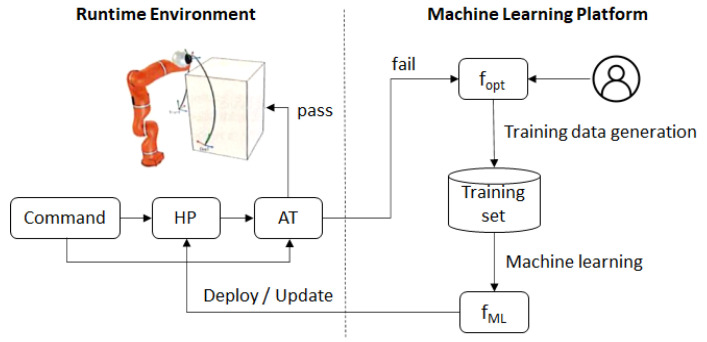
ML model training and runtime adaptation of the HP planner. *f_opt_*: an optimal computational motion planning algorithm, *f_ML_*: the procedure by which a ML model is first trained, and AT: acceptance test.

Figure 3 illustrates the procedure by which a ML model (denoted as *f_ML_*) is first trained using an optimal computational motion planning algorithm (denoted as *f_opt_*), then deployed as a HP planner, then retrained using an extended training set and updated during runtime. An expert generates a first training dataset using *f_opt_*. These data are used to train a first version of *f_ML_*, which is deployed as the HP planner. During operation, the input cases that fail the AT are collected and sent to the Machine Learning Platform. For every new input case, *f_opt_* is run to produce a complete training record, which is added to the training set. Using the extended training set, a new *f_ML_* is retrained, e.g., at regular intervals or whenever the amount of new input cases justifies retraining. Over time, the reliability of *f_ML_* improves, and the retraining process can be suspended when the reliability of the model has reached a satisfactory level. Retraining can be resumed when the system encounters a new situation that requires it to continue learning.

In the next section, the main features of the ASA are illustrated based on a dynamic path planning application with obstacle avoidance.

## 4. Application

**Figure 4:**
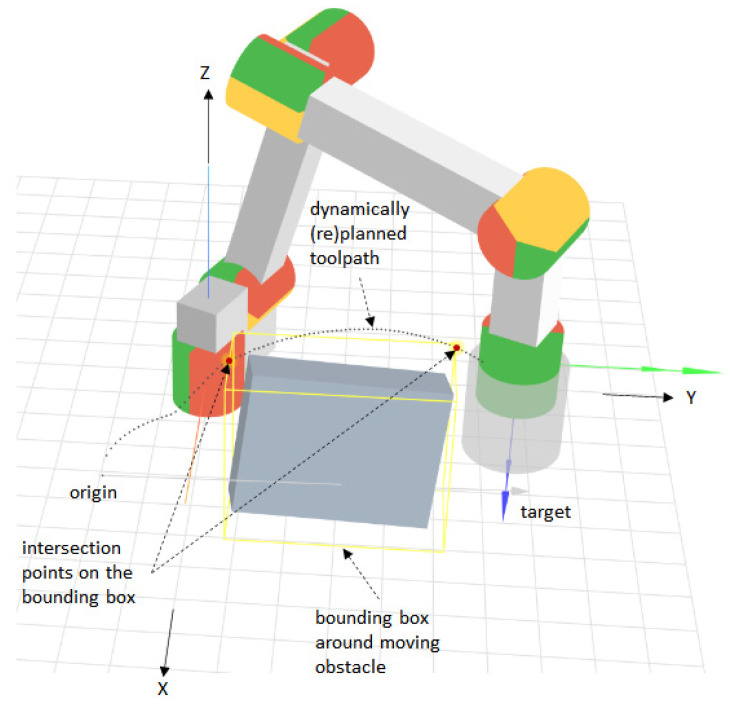
Dynamic path planning with obstacle avoidance.

To evaluate the adaptive simplex architecture, a dynamic path planning application with obstacle avoidance for a 6-DOF manipulator is considered (see Figure 4).

The moving obstacle can take arbitrary shapes and be composed of one or more distinct objects. It is assumed that a 3D sensor generates a point cloud around which a 3D-bounding box (or bounding volume) aligned with the axes of the robot’s base coordinate system is computed. The point cloud and the bounding box are updated in real time to facilitate collision avoidance through fast path replanning. The path is computed in the robot’s base coordinate system with respect to the robot’s tool center point (TCP). Given an origin and a target point, the problem consists of finding a smooth toolpath that avoids a moving object while minimizing the length of that path. Smooth toolpaths facilitate the computation of smooth trajectories, which help to mitigate joint stiffness and overshoot, and to reduce extra strain on robot actuators [41]. Shorter paths also reduce wear and energy consumption. Some authors note that path smoothness is a factor in the social perception of robots, with smoother paths being more acceptable [42].

To solve this path planning problem, an analytic solution based on Lamé curves is considered. Within the scope of this work, the advantage of an analytical approach, which can be formulated as a parameter optimization problem, over a heuristic, graph-based approach is that the former facilitates a simple formulation as a machine learning problem with practical applicability in human–robot collaboration scenarios. 

**Figure 5:**
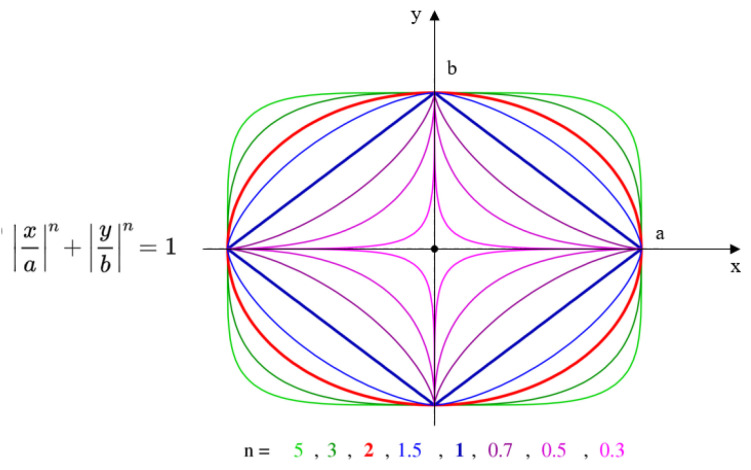
Lamé curve equation and various shapes the curve can take.

Figure 5 shows the Lamé curve equation and the different shapes that it can take, depending on the *a*, *b*, and *n* parameters. Assuming that the path from an origin point to a target point in the robot’s base coordinate system intersects an obstacle (see Figure 4), the obstacle avoidance problem with path length minimization can be formulated as:

(1) $$\min\left(\sum_{i=1}^{N-1}\sqrt{{\left({x^{\prime }}_{i+1}-{x^{\prime }}_{i}\right)}^{2}+{\left({y^{\prime }}_{i+1}-{y^{\prime }}_{i}\right)}^{2}}\right),\;\mathrm{where}\;{x^{\prime }}_{i}=b{\left(1-{\left|\frac{{x^{\prime }}_{i}}{a}\right|}^{n}\right)}^{\frac{1}{n}},{x^{\prime }}_{origin}\leq {x^{\prime }}_{i}\leq {x^{\prime }}_{target}\;\mathrm{subject}\;\mathrm{to}\;{\left|\frac{x^{\prime }}{a}\right|}^{n}+{\left|\frac{y^{\prime }}{b}\right|}^{n}<1,\;\left(x^{\prime },y^{\prime }\right)\in B.$$

**Figure 6:**
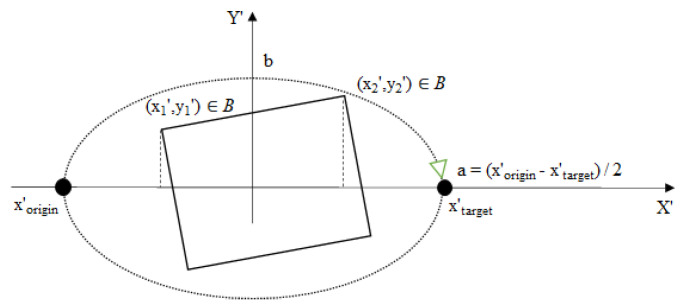
Section through the bounding box from Figure 4, defined by the orthogonal plane (with respect to the XY plane) containing the origin–target vector.

In Equation (1), *N* denotes the number of discrete segments along the elliptical path between the origin and target, as illustrated in Figure 6, and *B* denotes a set of representative points on the bounding box, as shown in Figure 6. The representative points on the 2D-bounding box in Figure 6 coincide with the intersection points on the 3D-bounding box illustrated in Figure 4. In Figure 6, when the obstacle is avoided in a vertical plane, the *Y′*-axis corresponds to the *Z*-axis in the robot’s base coordinate system. In general, the coordinate system in Figure 6 corresponds to the plane in which the curve is drawn, with the origin and target points lying on the *X*-axis of that coordinate system.

As the obstacle moves, the Lamé curve parameters *b* and *n* have to be recomputed in real time so as to satisfy Equation (1). To this end, at every step *i*, with 1 ≤ *i* < *N*, a new Lamé curve is generated, which satisfies the condition that all representative points of the bounding box be inside the curve. As a result, the toolpath will adapt to the current position and size of the bounding box around the obstacle.

### 4.1. Optimal Algorithm for Determining the Lamé Curve Parameters

Given the input set *I* = (*x′_origin_*, *x′_target_*, *x′*_1_, *y′*_1_, *x′*_2_, *y′*_2_), which describes the current position of the TCP, the position and size of the obstacle, and the target in the section plane from Figure 6, an optimal computational solution to the minimization problem Equation (1) can be implemented using a backtracking algorithm (see Algorithm 1). This algorithm iterates through all discrete combinations of the *b* and *n* parameters of the Lamé curve, which do not violate the inequality in Equation (1), and computes the arc length resulting from each solution. The inequality is checked in the function test, and the arc length is computed in evalArc. These functions do not take as parameters the origin and target of the motion, because Algorithm 1 operates with *x′_origin_* = 0 and *x′_target_* = 100, whereas *x′_origin_* and *x′_target_* are scaled during runtime to match the origin and target of the planned robot motion.

For small *b_step* and *n_step* values (e.g., 0.1 or lower), Algorithm 1 produces nearly optimal solutions with respect to the arc length, albeit at the cost of a high running time. Therefore, Algorithm 1 is run with small values for *b_step* and *n_step* only on the machine learning platform to produce training data. For small step values, Algorithm 1 corresponds to *f_opt_* in Figure 3. When Algorithm 1 is used as the HA planner, the two-step parameters need to be set to larger values (e.g., *b_step* = 2 and *n_step* = 0.5) in order to keep the running time low and, thus, to prevent jerky movements.
**Algorithm 1: Lamé curve parameter optimization.****Input:***x*′_1_, *y*′_1_, *x*′_2_, *y*′_2_, *b_step*, *n_step***Output:***b*, *n*best_*b* = −1, best_*n* = −1, best_arc = 10^4^, *n* = 10**do***n* = *n* − *n_step**b* = max(*y*′_1_, *y*′_2_) + 50**do**b = b − b_step*t* = *test*(*x*′_1_,*y*′_1_,*x*′_2_,*y*′_2_,*b*,*n*)**if***t* < 1arc = evalArc(*b*,*n*)**if** arc < best_arc best_arc = arcbest_*b* = *b*best_*n* = *n***while***b* > max(y′_1_, y′_2_) **and** *t* < 1**while***n* > 1**return** best_*b*, best_*n*

### 4.2. Machine Learning Model

Artificial neural networks (ANN) have been shown to approximate polynomial objective functions accurately [43,44]. This facilitates the training of ANN models to predict solutions to linear and nonlinear optimization problems using data generated by heuristic or exact algorithms designed to solve such problems [43,44]. Drawing on this theoretical result, a feedforward ANN can be trained with *I* = (*x′_origin_*, *x′_target_*, *x′*_1_, *y′*_1_, *x′*_2_, *y′*_2_) as the input and *O* = (*b*,*n*) as the output to solve Equation (1). For every input *I*, the computational algorithm described in the previous section can be used to find the best combination of *b* and *n*. To simplify the problem, *x′_origin_* and *x′_target_* can be set to 1 and 100, respectively, since the Lamé curve can be scaled on the *X*′-axis to any distance between the origin and target. The resulting model is able to predict a near-optimal solution to Equation (1) in real time.

The architecture of the feedforward ANN used to predict the parameters of the Lamé curve is as follows: 4 input and 2 output “min–max” normalized floating point numbers; 4 hidden layers with 32, 32, 32, and 16 neurons, respectively, and the Sigmoid activation function. This architecture was iteratively constructed by following the “funnel” rule, which states that the number of neurons per hidden layer should decrease from the input towards the output. Experiments have shown that, when fewer hidden layers and neurons are used, the network tends to overfit on the training data. The size of the network was gradually increased until the baseline model reached a satisfactory level of performance in terms of reliability and accuracy during testing (see Section 5.4. for detailed results).

**Figure 7:**
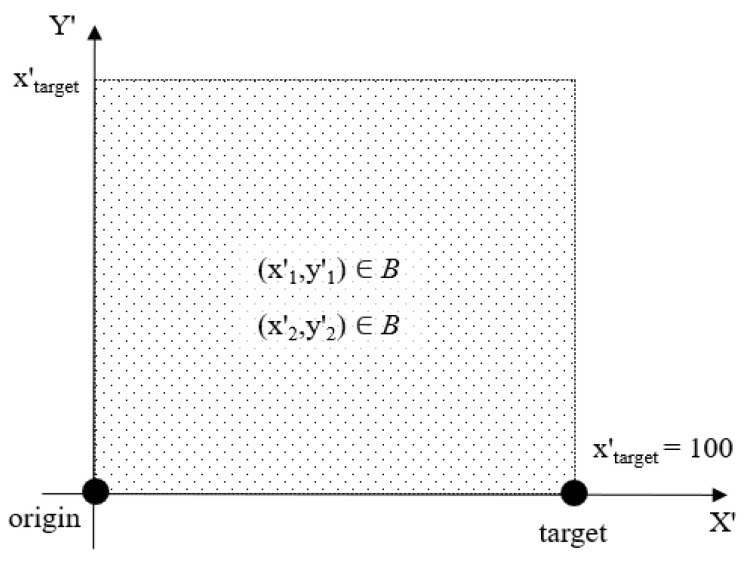
Range of the uniformly distributed baseline training data. The baseline data covers the entire problem space. Compared to Figure 6, the *X′*-axis is translated to the right by (*x′_target_*/2).

The model was initially trained using 10,000 input cases (denoted the baseline training set), whereby K-fold cross-validation [45] with *k* = 3 was used. The K-fold cross-validation method automatically divides the data into a training and a validation set. The parameter *k* determines the number of records used for training and validation, as well as the number of training cycles. For *k* = 3, 67% of the records are used for training and 33% for validation, which are sampled randomly from the data. The training procedure was repeated multiple times with different random divisions of training and validation records until either the 0.0006 training error or the 5000-epoch threshold was reached in each individual training cycle. The range of the input variables, which were all distributed uniformly, was chosen as follows: *x′*_1_ within *[1100]*, *x′*_2_ within *[x′*_1_, *100]*, and *y′*_1_, *y′*_2_ within *[1100]* (see Figure 7). For each of the 10,000 randomly generated, uniformly distributed combinations of points (*x′*_1_, *y′*_1_) and (*x′*_2_, *y′*_2_), the *b* and *n* parameters of the Lamé curve were computed using Algorithm 1. The training data, including the Lamé curve parameters, were “min–max” normalized, with *min* = 1 and *max* = 100. The resulting model was used as the initial version of the HP planner in the ASA.

### 4.3. Acceptance Test

The acceptance test (AT) used to decide whether the pair of points (*x′*_1_, *y′*_1_) and (*x′*_2_, *y′*_2_) are inside the Lamé curve characterized by the parameters (*b*, *n*) is facilitated by the inequality from Equation (1). If the inequality holds for a given pair of points in the section plane from Figure 6, the test verdict is “pass”; otherwise, it is “fail”. This acceptance test is used both as part of Algorithm 1 and to check the prediction of the ML model. The proposed AT performs a constant number of mathematical operations, which makes it very fast.

### 4.4. Runtime Adaptation

The adaptation of the ML-model used as the HP planner (i.e., *f_ML_* in Figure 3) requires monitoring its inputs and outputs and the verdict of the AT in an operational setting. The inputs for which the model’s outputs fail the AT are added to the training set together with the corresponding optimal Lamé curve parameters, *b* and *n*, computed using *f_opt_*. Since, in an operational setting, some failures are likely to occur on similar input cases, redundant (i.e., identical or very similar) inputs must be removed from the training set to avoid overfitting. This can be achieved by filtering out very similar inputs.

**Figure 8:**
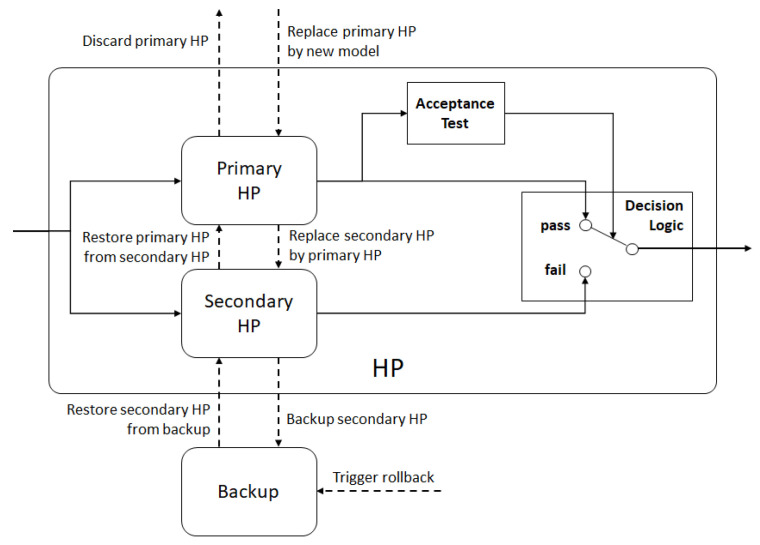
HP planner container implemented as a recovery block.

The HP planner is updated whenever a new ML model is available. Since data generation is continuous, the model training process can be configured to update the HP planner at regular intervals. When a new model is deployed as the HP planner, there is a likelihood that it will perform worse than the current model. To prevent the performance degradation of the ASA, the HP planner can be implemented as a recovery block [46], as shown in Figure 8. When a new model becomes available, it is deployed as the primary HP, whereas the then–current primary HP replaces the secondary HP, which is stored for backup. If the new model performs worse than the secondary HP (i.e., the former primary HP), then a rollback is triggered, which restores the primary and secondary HPs as they were before the new model was deployed. This strategy helps to automate the creation and deployment of a new ML model as the HP planner without degrading the overall performance of the ASA.

Adapting the ML model used as the HP planner amounts to a problem of continual learning [47] without “catastrophic forgetting” [2]. Continual learning techniques aim at retraining ML models so as to include new data without degrading the performance of the original model. In reference [2], p. 3521, “catastrophic forgetting” is defined as “the tendency for knowledge of the previously learned task(s) (e.g., task A) to be abruptly lost as information relevant to the current task (e.g., task B) is incorporated”.

In the case of the Lamé curve parameter approximation problem, the original training set already covers the entire problem space (i.e., the dotted square in Figure 7). However, models trained using the uniformly distributed input set have lower performances at the boundary of the problem space—i.e., they fail the AT more often for obstacles that are close to the boundary of the problem space. This is because, close to the boundary, the training data are biased towards the interior of the problem space, since there are no data outside that space. This prevents the ANN from learning how to correctly estimate *f_opt_* at the boundary of the problem space. This issue can be overcome by extending the problem space and projecting the origin and target at a “safe” distance from the boundary. This, however, requires a significant redesign of the entire solution approach. The ASA proves its usefulness in situations when such a redesign is not possible or undesirable by facilitating continual learning from failure. Instead of redesigning the solution approach, the ML model is retrained using the data collected during the operation.

## 5. Evaluation

This section presents an evaluation of the proposed approach inspired by two human–robot collaboration scenarios in manufacturing. After providing some background on the safety issues related to HRC in manufacturing, the two scenarios are described. The ASA for dynamic obstacle avoidance is evaluated in view of the practical implementation of these scenarios. The approach is first evaluated using a simulated redundant 6-DOF manipulator. The robot toolpath follows a Lamé curve around a dynamically computed 3D-bounding box, which can contain one or several objects. To evaluate the practical feasibility of the approach, the Lamé curve-based path planning method was implemented for a Universal Robot 5 and evaluated experimentally in the Industry 4.0 Pilot Factory of the Vienna University of Technology, Vienna, Austria [48].

### 5.1. Human–Robot Collaboration Safety

Collaborative robot systems can be operated without a safety fence in the proximity of human workers, provided that every application undergoes a risk assessment according to the current safety norms and standards. In addition to the country-specific machinery directives, specialized standards and regulations for robot safety—in particular, ISO 15066 [49] and 10218 [50] (industrial robot safety requirements)—specify the regulatory framework for safe cooperation between humans and robots in an industrial context [51]. Collaboration is one of the four modes of human–robot interactions defined by the ISO 10218 standard and requires the strictest risk assessment. In human–robot collaborations, robot systems act as the physical interfaces of digitized production, which reach into the work environment of humans. A first condition for the certification of a collaborative human–robot application is the use of a specially designed collaborative robot (e.g., Universal Robot 3/5/10, KUKA iiwa, etc.). A collaborative robot is endowed with highly reliable sensors and functionality, which allow the robot to continuously monitor and limit its motion velocity. Such functionalities form the basic building blocks for the implementation of the four possible collaborative operating modes foreseen by the ISO 10218 standard for an industrial robot: safety-rated monitored stop, hand guidance, speed and distance monitoring, power, and force limitation [51]. Although the speed and distance monitoring mode can potentially provide effective safety features in scenarios where direct physical contact between humans and robots is required, the current (3D) sensor technologies in combination with complex machine learning techniques are not yet capable of fulfilling the requirements of the ISO 10218 standard [51]. In this context, the proposed ASA for dynamical obstacle avoidance aims to advance the state-of-the-art in safe, reliable, and robust machine learning techniques for flexible collaborative human–robot applications with speed and distance monitoring.

### 5.2. Application Scenarios

**Figure 9:**
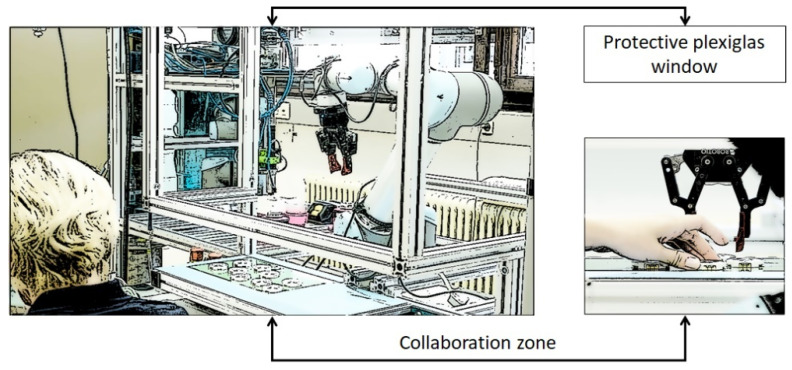
Plexibot scenario: human–robot collaboration (HRC) application from bayerdynamic^®^ [53].

We consider two human–robot collaboration scenarios that are often encountered in industrial applications. In the first scenario (denoted Plexibot), a collaborative robot arm is operated behind a framed Plexiglas window with a ~20-cm opening in the lower part, as shown in Figure 9 [52]. Through the opening, a worker can manipulate work pieces in the same region as the robot. This assembly station layout allows higher robot speeds than a layout in which the robot is unconfined, because the worker’s head is out of the robot’s reach. Upon (accidently) colliding with the human hand, the robot automatically stops.

The ASA aims to improve on this solution by preventing collisions with the worker’s hands, thus reducing the likelihood of a safety stop, which would require human intervention to unlock the robot. The 3D camera, which is already mounted on the robot arm, can be used to generate a point cloud representing all the obstacles that are in the robot’s way, including the worker’s hands. This reduces the eventual downtimes resulting from recovering from safety stops while increasing the workers’ confidence in the robot and the acceptability of collaborative robots in manufacturing. To ensure the workers’ safety at all times, the robot toolpath follows a parameterized Lamé curve in the vertical plane with respect to the assembly table.

**Figure 10:**
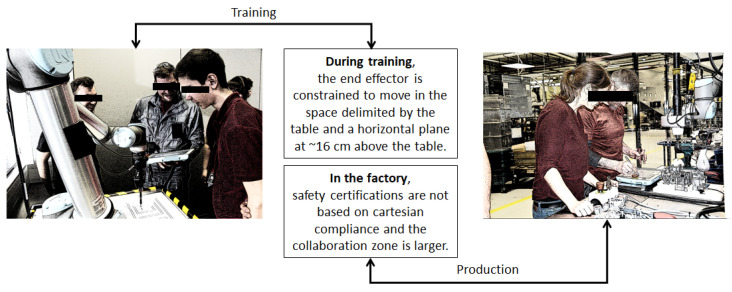
Edubot scenario: Universal Robot (UR) 10 collaborative robot used for education and training purposes in a manufacturing company with trainer and trainees [53,54].

In a second scenario (denoted Edubot—see Figure 10) [53,54], the focus is on a manufacturing company that runs a robot operator training center. To ensure safety during training, the company hired a specialized consultancy company to conduct a safety risk assessment for a HRC application for educational purposes involving a Universal Robot 10. Given that no robot confinement structure was used during training and that teach-in-based programming and testing were performed by novice users, the risk assessment recommended that the movement range of the 6-DOF robot be constrained to prevent the robot’s end effector from reaching higher than about 16 cm above the training table and, thus, to prevent any collisions with the trainees’ upper body and head. In addition, the orientation of the end effector was constrained to always point downward, and the speed of the end effector was limited to 150 mm/s. One problem that emerges from these restrictive safety settings is that trainees learn how to use the robot in this Cartesian-compliant mode only to be faced with a completely different situation in productive applications, which are not subjected to the same cartesian compliance rules, provided that all robot movements and positions are predefined and fixed so that the risk assessment can be conducted based on force and momentum measurements. In this context, the manufacturing company seeks to align the safety principles used during training and the factory settings so as to facilitate the trainees a smoother transition from educational to productive robot use.

**Figure 11:**
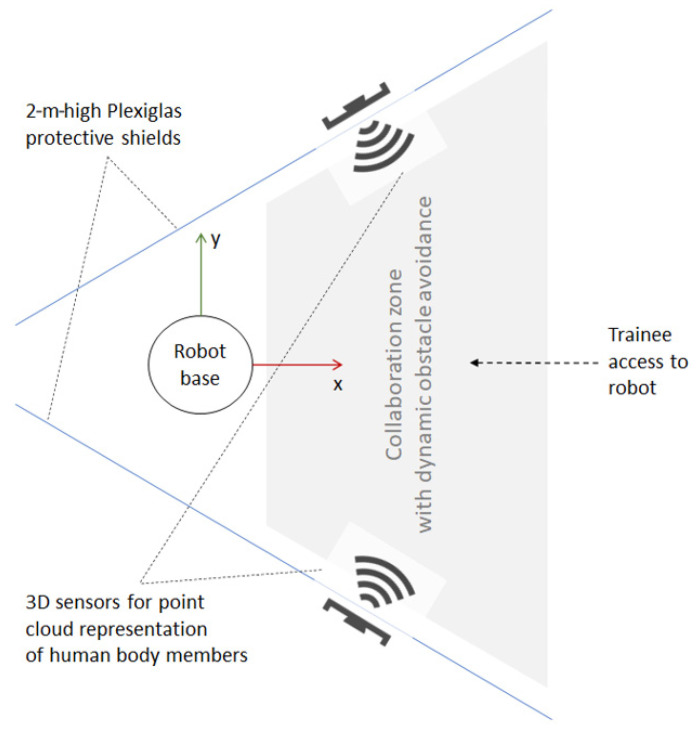
Edubot scenario: Proposed layout for the training robot station.

The ASA aims to improve on this solution by providing a fast and reliable dynamic obstacle avoidance solution for the training setting so that the 16-cm limit can be gradually increased, depending on the experience of the trainees, until it can be eliminated completely. For this purpose, the robot station can be redesigned, as shown in Figure 11. The robot is partly confined by two Plexiglas protective shields on the sides so that only the trainee who currently works with the robot can access it from one side, while the other trainees can watch the robot in motion from behind the shields. The collaboration zone is surveilled by two 3D sensors that are able to detect and trace the trainees’ hands and other body parts. The 3D sensors generate point clouds, around which bounding boxes can be computed as inputs to the path planner. The grey trapezoid in Figure 11 is a table at ~1 m above the ground. Using dynamic obstacle avoidance during program testing, the constraints resulting from the initial risk assessment can be gradually lifted as the trainees gain more theoretical knowledge and practical experience.

In the following, the ASA is evaluated for dynamic obstacle avoidance in the vertical and horizontal planes in view of the practical implementation of the Plexibot and Edubot scenarios, respectively.

### 5.3. Simulation-Based Implementation

**Figure 12:**
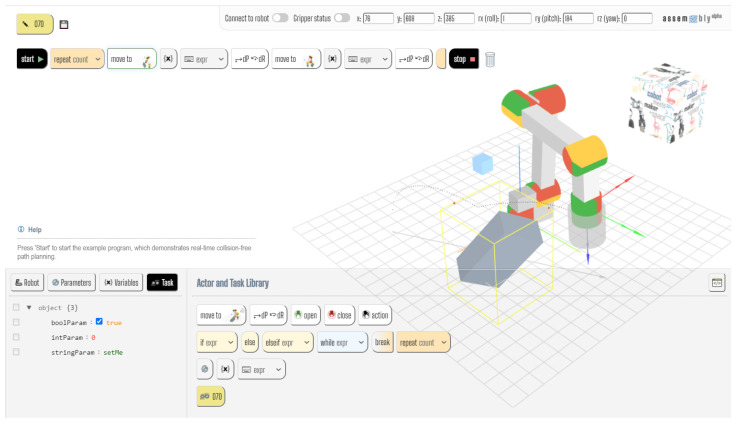
Generic robot arm visualization with a rotating cuboid obstacle in the Assembly [4,5] robot programming and simulation environment.

The performance of the ASA was evaluated using a web-based generic robot programming and simulation tool called Assembly [4,5] (see Figure 12). This tool provides a mixed textual/graphical block-based program editor and integrates an open source robot arm simulator (available online: https://github.com/glumb/robot-gui) (accessed on 21 March 2021) developed in Three.js (a JavaScript animation library based on WebGL, available online: https://threejs.org (accessed on 21 March 2021)). In this environment, a moving obstacle in the form of a translating and rotating cuboid was created, as shown in Figure 4. The size of the virtual cuboid is 15/45/25 cm, which covers most human hands and lower arms in various positions. While the obstacle rotates around all three axes by ~4.5° per s, a bounding box aligned with the X-, Y-, and Z-axes of the base coordinates system is computed and displayed around it. The coordinates of this box are used to determine the characteristic points (*x′*_1_, *y′*_2_) and (*x′*_2_, *y′*_2_), as shown in Figure 4 and Figure 6. Figure 9 shows the robot simulator and the obstacle in the context of the Assembly programming and simulation environment. To train and score the different ANN models used during the evaluation, the Brain.js JavaScript-based ML library was used (available online: https://brain.js.org (accessed on 21 March 2021)).

To ensure real-time collision avoidance, each link of the robot was endowed with nine equidistant collision checkpoints, two of which were placed in the adjacent joints. During the motion, the planner checks at a frequency of 33 Hz whether any of the collision checkpoints finds itself within the bounding box around the obstacle. This enables a fast reaction by the motion planner whenever a potential collision with the obstacle is detected—in which case, the robot stops and waits until the obstacle moves away. Note that, in the considered human–robot collaboration scenarios, the obstacles are primarily represented by moving hands and other body members rather than fixed objects. In this context, it is assumed that the human will not willingly block the robot without a good reason (e.g., a safety hazard or a planned action); in which case, the robot must stop. It is also assumed that the human–robot collaboration applications are designed such that the origin and target poses are not impossible to reach (e.g., due to fixed objects).

**Figure 13:**
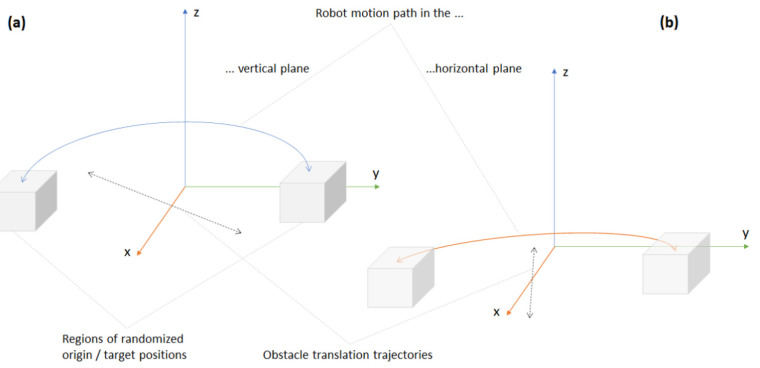
Sketch of the test configurations with dynamic obstacle avoidance in the (**a**) vertical and (**b**) horizontal planes.

The test program moves the robot arm between two randomized locations, which were chosen so that the path from the first (origin) to the second (target) location cross the obstacle’s bounding box. Before each odd run, the origin is randomized in the Y–Z plane, and, before each even run, on the X–Y plane, so that the test program can sample from about 20,000 random origin locations (see Figure 13). The orientation of the end effectors is also randomized, with *rx* ranging between [−50°, 50°] and *ry* between [130°, 230°]. To evaluate the ASA’s capacity to learn, the bounding box around the rotating obstacle is translated from one extreme of the problem space to another. As described in Section 4.4., the situations in which the obstacle finds itself close to the boundaries of the problem space challenge the ML model, which needs to be retrained and redeployed in order to improve the performance of the HP planner.

According to the procedure illustrated in Figure 3, after each test run, which comprised 60 robot movements between the origin and target, the input cases that failed the AT were added to the training set, and the ML model used as the HP planner was retrained and redeployed. Figure 13 illustrates the different test configurations used during the evaluation. Two test rounds were conducted—one with obstacle avoidance in the vertical plane, which corresponds to the Plexibot scenario, and another with obstacle avoidance in the horizontal plane, which corresponds to the Edubot scenario.

### 5.4. Results

#### 5.4.1. Reliability and Adaptability

**Figure 14:**
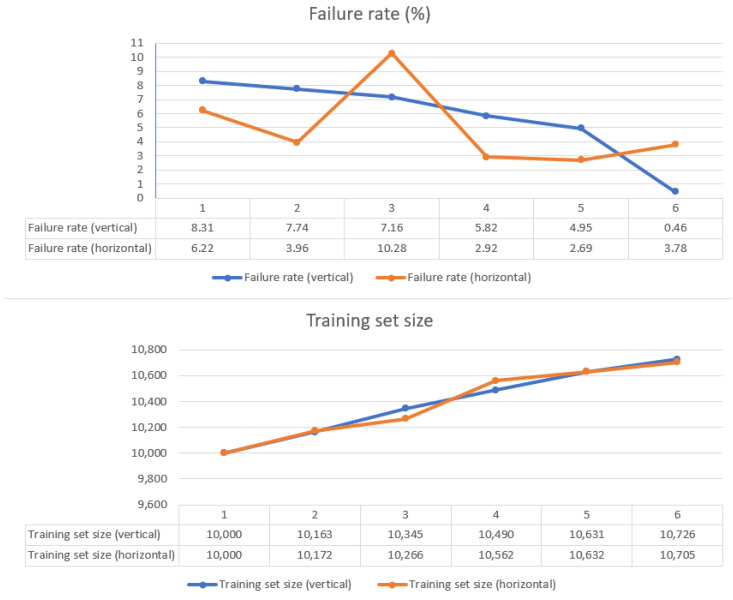
Top: The failure rates of the 6 different models used during the evaluation in the vertical and horizontal test configurations. Bottom: The size of the training sets used to train the 6 models.

Figure 14 shows the evaluation results of the two test runs (for the vertical and horizontal test configurations) in terms of the reliability of the different ML models used as the HP path planner and the adaptability of the ASA. For both configurations, the initial model was trained using the baseline training set described in Section 4.2., which contains 10,000 uniformly distributed input cases. Then, after each test cycle, which comprised 60 robot movements between randomized origin and target poses, the model was retrained using the baseline training set to which the input cases for which the model predictions failed the AT were added. The resulting training set was filtered to remove identical or very similar input cases. The result of a model run (or scoring) is considered to have failed if the obstacle is not contained within the Lamé curve characterized by the parameters *b* and *n*, as predicted by the model. In each test cycle, the model is scored 6000 times, because the path between the origin and target is divided into 100 segments.

The failure rate chart at the top of Figure 14 illustrates the performance of the 12 models that were tested (six for the vertical configuration and six for the horizontal configuration). In the vertical configuration (blue curve), the initial (baseline) model yielded a failure rate of 8.31%. The retrained models iteratively improved on this result. The sixth model achieved the best performance, with a failure rate of only 0.46%. In the horizontal configuration (orange line), a different pattern can be observed. Here, the fifth model achieves the lowest failure rate of 2.69%. The different patterns can be explained by the different obstacle translation trajectories and distributions of the origin/target poses used in the vertical and horizontal configurations (see Figure 13). Additionally, training ANNs is a stochastic process, in which the training set is shuffled in each epoch. This process can thus lead to different learning patterns. Nevertheless, extensive tests have shown that the proposed retraining strategy yields a very low failure rate after five–seven retraining cycles.

**Figure 15:**
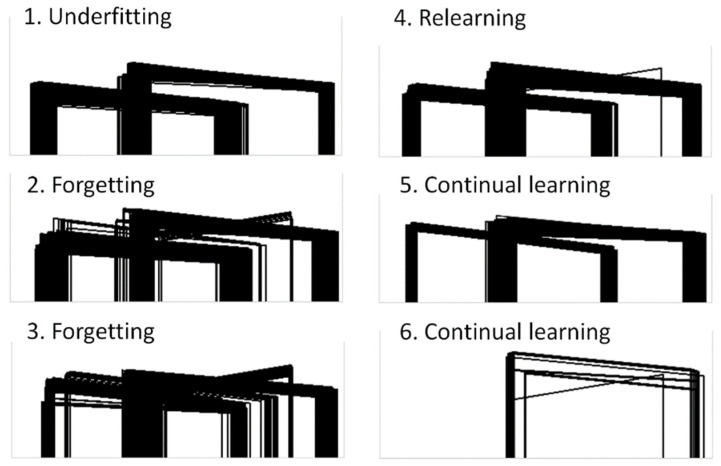
Obstacle silhouettes for which the 6 ML models used in the vertical test configuration failed the acceptance test.

Figure 15 illustrates the obstacle silhouettes for which the models used in the vertical test configuration failed the acceptance test. These silhouettes provide insights into how the ANN learns and/or forgets from one retraining cycle to another. The first (baseline) model was underfitted and therefore failed when the obstacle was close to the extremes of the problem space. After the first and second retraining cycles, models 2 and 3 slightly improved their failure rates at the cost of forgetting how to deal with some of the input cases that did not challenge the baseline model. After the third retraining cycle, model 4 seemed to relearn what was forgotten by models 2 and 3. Model 5 was the first to exhibit a clear improvement over the baseline model, whereas model 6 seemed to learn how to deal with the extreme cases quite well.

#### 5.4.2. Running Time, Path Length, and Path Smoothness

To compare the performances of ML-based HP planners with those of the optimal computational method (i.e., *f_opt_*) and of the fast heuristic (i.e., *f_fast_*) used as the HA planner, a series of tests with different ASA configurations were conducted. In the first ASA configuration (denoted *f_ML_* + *f_opt_*), the best performing model in the vertical test configuration (i.e., model 6 in the previous set of tests) was used as the HP planner and Algorithm 1 with *b_step* = 0.2 and *n_step* = 0.1 as the HA planner. Given these step sizes, Algorithm 1 took about one second to determine the Lamé curve parameters (*b*, *n*) of the minimal arc length for a given obstacle configuration and could thus be considered very slow. In a second ASA configuration, denoted as *f_ML_* + *f_fast_*, with *b_step* = 2 and *n_step* = 0.5, Algorithm 1 became a fast, yet less accurate, heuristic compared to *f_opt_*. To provide an objective baseline for comparison, the two versions of Algorithm 1 were also considered as standalone path planners (configurations denoted as *f_opt_* and *f_fast_*).

The running time, path length, and path smoothness were measured for 60 robot movements between the origin and target using the vertical configuration from the previous set of tests. Between the origin and target, the ML model is scored 100 times in order to dynamically adapt the movement trajectory to the moving obstacle’s position. The planning time per test run, *t_p_*, is measured as the average time required by the respective planner to determine the Lamé curve parameters during one robot movement between the origin and target, which requires 100 replanning cycles. The waiting time per test run, *t_w_*, is measured as the total waiting time due to potential collisions with the robot’s joints or links divided by the total number of test runs (i.e., 60 in the current test configuration). The motion time per test run, *t_m_*, is the total time that it takes the robot to move between the origin and target while dynamically replanning the path and avoiding collisions.

To account for the different obstacle positions and orientations in measuring the path length, a so-called path factor was computed. The path factor is expressed as *p_f_* = *L/L_min_*, where *L* is the average length of the obstacle avoiding the path travelled by the robot’s TCP between the origin and target, and *L_min_* = *dist* (*origin*, *p*_1_) + *dist* (*p*_1_, *p*_2_) + *dist* (*p*_2_, *target*), where *p*_1_ = (*x′*_1_, *y′*_1_) and *p*_1_ = (*x′*_2_, *y′*_2_), as defined in Figure 6. *L_min_* thus represents the shortest collision-free path, which is impractical for moving obstacles, because it “touches” the object.

To measure the smoothness of the robot’s toolpath along the Lamé curve, a so-called smoothness factor based on reference [42] was used. The Lamé curve parameters are recomputed 100 times per movement between the origin and target, and each path segment represents a linear approximation of the corresponding Lamé curve arc segment. Depending on the ASA configuration used and on the obstacle’s dynamics, the robot continuously switches from one curve trajectory to another. If two subsequent Lamé curves differ significantly one from another in terms of *b* and *n*, then the robot will visibly “jump” from one curve to another, which leads to unsmooth movements. The smoothness factor (denoted as *s_f_*) used in this evaluation is defined as the sum of the angles (in radians) between the linear path segments along the Lamé curve with recomputed parameters divided by the length of the respective segment.

Table 1 summarizes the results of this evaluation. The results suggest that the two ASA configurations outperform the other two configurations in terms of time and smoothness. In terms of planning time (*t_p_*), the *f_ML_* + *f_fast_* configuration is ~1000 times faster than *f_opt_* and ~20 times faster than *f_fast_*. This suggests that the ASA can be used to replan the path up to ~3000 times per second on an Intel i7 processor compared to ~128 times when *f_fast_* is used. At the same time, the two ASA configurations both outperformed *f_fast_* in terms of the path length. *f_opt_* yielded the shortest average path, as expected. In terms of path smoothness, the two ASA configurations clearly outperformed the other two configurations.

Two videos illustrating the approach in the vertical and horizontal test configurations are available online [55,56]. The two videos illustrate the performance of the *f_fast_* configuration on the left-hand side and that of the *f_ML_* + *f_fast_* configuration on the right-hand side. The *f_ML_* + *f_fast_* configuration produces visibly smoother and, consequently, shorter paths than the *f_fast_* configuration. In the horizontal configuration (second video [56]), the *f_fast_* planner leads to more situations in which the robot waits for the obstacle to move away after a collision is detected. By contrast, thanks to its superior performance in terms of path smoothness and speed, the *f_ML_* + *f_fast_* planner helps to minimize the waiting time.

### 5.5. Comparative Performance

The performances of the proposed approach were compared with those of two ML-based methods [26,27], the development of which was driven by the requirements of human–robot collaboration, an efficient model predictive control (MPC)-based planning algorithm for 6-DOF manipulators with dynamic obstacle avoidance [31], and the popular Rapidly-exploring Random Trees (RRT) Connect [57] algorithm, which is integrated in the Robotics Library [58] and other open-source motion planning libraries. The first ML-based method, called Soft Actor-Critic with Hindsight Experience Replay (SAC–HER) [26], builds on reinforcement learning to plan motion paths for a dual-arm robot, with each arm having 3-DOF. The planning occurs jointly for both robots, as if they were part of the same entity. As a result, the planning is performed for 6-DOF. The second Soft Actor-Critic (SAC)-based method [27] extends the SAC–HER approach by using a dual-arm robot, with each arm having 7-DOF. The MPC-based algorithm [31] uses the distance between any of the six robot joints and one or several moving obstacles to plan a feasible trajectory for a 6-DOF robot. The method was tested using a UR 10 robot with moving objects and a full-scale human model. Although this method does not use machine learning, it is capable of real-time trajectory planning with dynamic obstacle avoidance.

**Figure 16:**
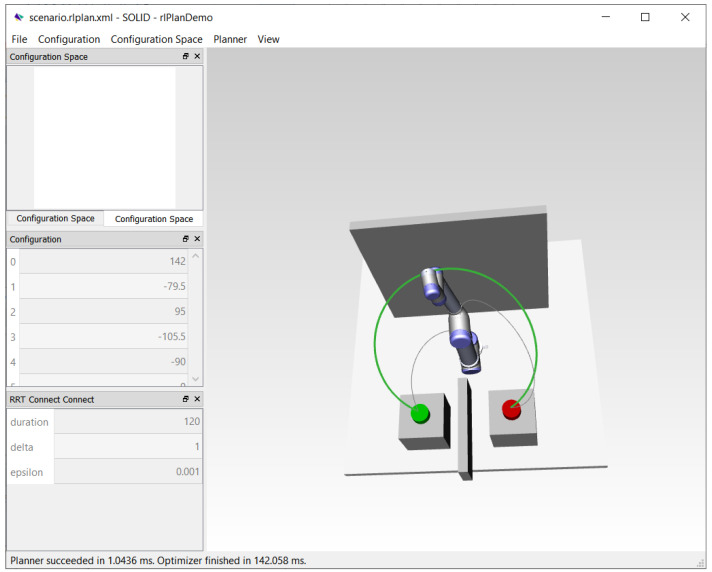
Screen capture of a Robotics Library [58] simulation with a UR 5 six degrees of freedom (6-DOF) robot following a path generated by the RRT Connect algorithm [57].

To create a baseline for comparison, the experimental results from references [26,27,31], which provide information about the performances of the respective planning methods, were drawn upon. For the RRT Connect planner, an experiment in the Robotics Library [58] simulation environment was conducted. To this end, a scenario similar to the one used for testing the proposed approach (albeit with a fixed obstacle) was reconstructed in the simulation environment (see Figure 16).

Table 2 reports the results of this comparison. The performances of the ASA (*f_ML_* + *f_fast_*) configuration reported in Table 1 were compared to those of the other four algorithms, as they were reported in their respective papers. In Table 2, the replanning time is the time it takes the respective planner to produce a motion plan repeatedly while the robot is moving. For the methods that do not consider dynamic obstacle avoidance, planning occurs once, and therefore, the replanning time is the same as the planning time.

In Table 1, we reported the planning and waiting times per robot movement (between the origin and target) for the different ASA configurations. This time represents the sum of 100 replanning cycles, which are performed during one such movement. Therefore, in Table 2, the replanning time is computed as (*t_p_* + *t_w_*)/100. The replanning times reported for the MPC-based method in reference [31] allow for up to 100 replanning cycles per second, whereas the ASA can achieve up to 1000 replanning cycles per second. When a human enters the scene, the measurements reported in reference [31] suggest that the MPC-based method is able to replan the path up to ~25 times per second (41.1 ms per replanning cycle). The SAC–HER method appears to be slower than the ASA and MPC. The authors of the SAC-based method for the two 7-DOF robot arms did not report any time-related performances. The path overhead was low for all the methods, except for RRT Connect. As Figure 16 shows, the RRT Connect planner was not only the slowest in the comparison but also inefficient in terms of path length. Concerning the success rate, the ASA and MPC have 100% planning success when waiting is used. According to the authors of reference [31], the MPC-based method gets stuck in local optima; in which case, the robot waits for the human to move away. This approach is similar to that used by the ASA. Compared to the other two ML-based models, the ASA uses a much smaller neural network. Compared to all the other models, the implementation complexity of the ASA is low when considering the theory behind each of these methods.

### 5.6. Experimental Validation Using a Collaborative 6-DOF Robot

**Figure 17:**
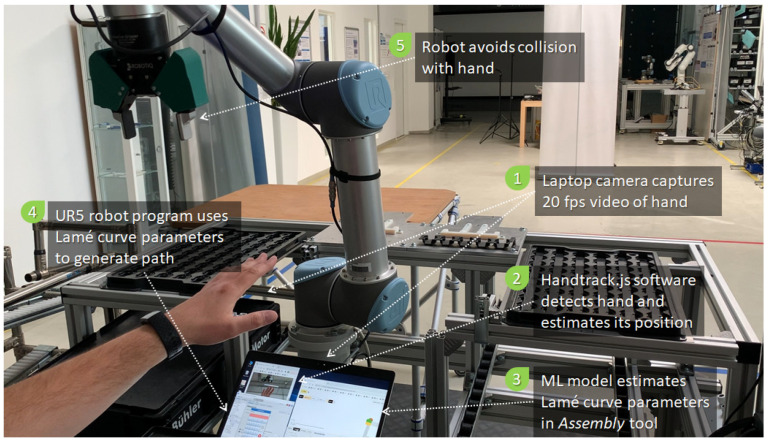
Experimental setup in the Industry 4.0 Pilot Factory of the Vienna University of Technology (fps = frames per second).

To demonstrate the feasibility of the approach, the simulation-based ASA implementation in Assembly was coupled with a UR 5 (CB series) robot arm in the Industry 4.0 Pilot Factory of the Vienna University of Technology, Vienna, Austria [48]. Figure 17 illustrates the experimental setup, which is based on the Flexibot scenario.

A program that uses the Lamé curve equation to generate an elliptical toolpath between two randomized poses was implemented in the robot’s native programming environment, called Polyscope. The robot program is coupled with the Assembly environment, where the ML model is used to estimate the Lamé curve parameters. For the purpose of this experimental validation, a simple visual hand tracking library called Handtrack.js (available online: https://github.com/victordibia/handtrack.js/ (accessed on 21 March 2021)) was used to estimate the position of the hand. Based on that information, a bounding box was computed around the hand in the simulation environment. This facilitated the computation of the Lamé curve parameters. The communication between the Assembly simulation environment and Polyscope was implemented using remote procedure calls over the XMLRPC protocol. The robot program polled the XMLRPC service provided by Assembly every 100 ms. This service returned the Lamé curve parameters corresponding to the current size and position of the bounding box.

**Figure 18:**
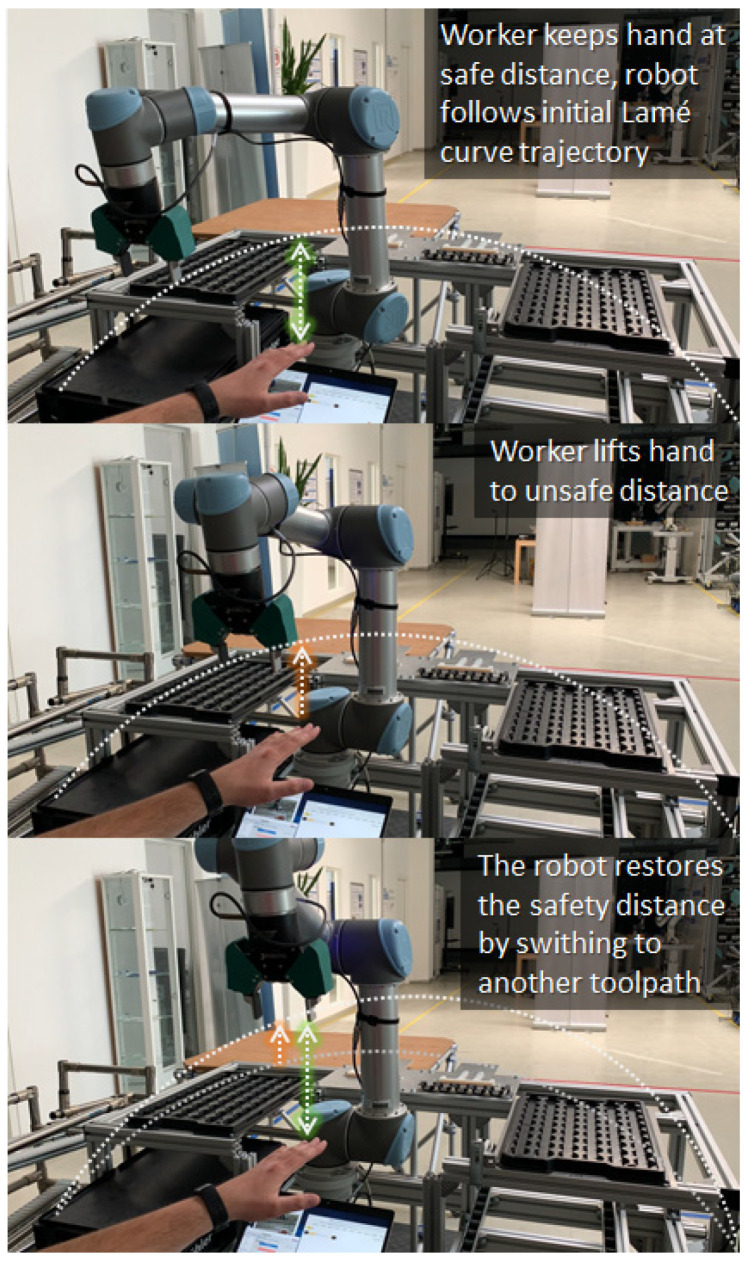
Robot avoids collision by adapting the toolpath to the position of the moving hand.

Figure 18 illustrates the dynamics of the experiment. As the robot moves between two arbitrary poses, it reacts to the position of the moving hand by adapting the current toolpath. The following video illustrates this behavior: reference [59]. For safety reasons, the speed of the robot was limited to 250 mm/s. Note that the communication over XMLRPC induced latency, which can be avoided by using a hardware-based protocol, like MODBUS. Additionally, the precision and performance of the Handtrack.js library are average. Higher performances can be obtained using a state-of-the-art 3D sensor and a fast object detection method. Using these technologies, the robot can operate at higher speeds without posing any safety risks.

## 6. Discussion

Concerning the reliability and adaptability of the ASA, the evaluation results suggest that, by simply adding the input cases for which the different model versions fail the AT, after only four to five retraining cycles, the ANN is learning continually, which significantly reduces the retrained model’s failure rate. This strategy does not require human intervention for modifying the architecture of the network or adjusting any other parameters of the training process. The ASA thus proves that it can adapt to new situations that were not covered by the baseline training set. In combination with the recovery block configuration from Figure 8, the failure rate of the HP planner in the ASA will stabilize that of the best performing model, even if the performance of the retrained model deteriorates. Alternatively, the decision to retrain the model can be taken on the basis of its historical failure rate. The failure rate of a model that performs well in some situations is likely to increase again when it is faced with a new geometric configuration or when the obstacles are closer to another boundary of the problem space.

Concerning the running time, path length, and path smoothness, the evaluation results suggest that ASA enables much faster and smoother path (re)planning than the computational methods for determining the Lamé curve parameters at the cost of a 6.74% longer path with respect to *f_opt_*. This enables the ASA to react very fast to moving obstacles, which is a prerequisite for safety assurance in human–robot collaboration.

In the Plexibot application scenario, the ASA (*f_ML_* + *f_opt_*) configuration with obstacle avoidance in the vertical plane can help to prevent any accidental collisions with the worker’s hands. This enables faster robot speeds, which fosters productivity without compromising safety. In this scenario, the robot is space-constrained by the Plexiglas shield, which justifies the use of *f_opt_* as the HA planner in order to keep the robot’s path close to the obstacle and, thus, to prevent collisions with the shield.

In the Edubot scenario, the ASA (*f_ML_* + *f_fast_*) with obstacle avoidance in the horizontal plane ensures the fastest reaction speed to the eventual interferences of the trainees with the moving robot. In the case of potential collisions, in this scenario, the robot always takes the path that is closer to the robot’s base in the X–Y plane. Hence, the trainees are protected from accidental collisions with the robot by the shields in the back and on the sides, and by the ASA in the front, without reducing the frontal collaboration zone. Thanks to the additional safety net provided by the ASA, some of the constraints resulting from the initial risk assessment (notably, the limitation of the range of the robot’s TCP in the vertical plane) can be lifted gradually, depending on the trainees’ experience.

The comparisons with other path planning methods from the literature suggest that the ASA (*f_ML_* + *f_fast_*) configuration provides a simple, practical, fast, and reliable path planning method for 6-DOF robots. This conclusion was substantiated by an experimental validation of the proposed path planning method, which was implemented for a UR 5 robot in an Industry 4.0 R&D environment.

### 6.1. Generalizability of the ASA

In this paper, the collision-free path planning problem with dynamic obstacle avoidance was considered as an example application of the ASA in robotics. In doing so, the problem was formulated as a combinatorial optimization problem that allows a parameterized analytic solution in the form of the Lamé curve. The exact (i.e., optimal) algorithm that optimizes these parameters for a given obstacle was used to generate training data for an ANN model, which was then used as the HP planner. The exact algorithm and a faster heuristic were then used as the HA planner. The ASA can thus be generalized to an entire class of problems in robotics, which (1) can be formulated as combinatorial optimization problems, (2) allow analytic or stochastic solutions with a fixed set of input and output parameters, and (3) allow an acceptance test that can reliably assert the safety of the HP planner’s predictions. In this sense, the ASA can, for example, also be used in combination with existing ML-based approaches for the fast, inverse kinematic calculations of various redundant manipulators (e.g., [60,61,62,63]) or for the ML-based corrections of the Assembly part positions (e.g., [64]). In the former case, a reliable acceptance test is facilitated by the direct kinematics calculations, whereas, in the latter case, CAD models can be used to check if the output of the HP conforms to the expected part model. In path planning applications with or without dynamic obstacle avoidance, the ASA can also be used for fast and safe autonomous vehicle and drone navigation (see related works for references), harbor crane guidance [65], and autonomous underwater vehicle navigation [66].

### 6.2. Limitations and Future Works

The retraining of ANNs, as well as other types of ML models, requires more computational resources than a robot controller usually provides. Therefore, the robot needs to be connected to a computational environment, like an industrial cloud or edge device, where model training is carried out. This introduces security issues, the tackling of which is beyond the scope of this work. Once these security issues are properly addressed and the retraining can be carried out in the cloud, models trained using data from a certain robot can be deployed on other robots as well. This will significantly accelerate the learning process in new application contexts and dynamic operational environments.

In terms of the latency and replanning frequency, the performances of the ASA are contingent on the performances of the 3D sensors and point cloud processing techniques being used. A frame rate of up to 100 frames per second and 10-ms latency for point cloud generation are typical of the current commercial 3D cameras. The generation of bounding boxes around obstacles requires the processing of the point cloud, which adds another ~10ms of latency. One can thus expect a real-time performance of ~50 updates per second conditioned by a latency of ~20 ms with the currently available 3D sensor technology. An evaluation in a real factory involving hardware sensors is currently being planned.

For the purpose of evaluating the ASA, a bounding box was generated around the moving obstacle in real time. To overcome this limitation, the ML model used as the HP planner can be trained with a relevant sample of points from a convex hull around an object. This is likely to facilitate shorter and smoother paths, but it would require a larger ML model (i.e., more layers, neurons, and training data).

## 7. Conclusions

This paper introduced the adaptive simplex architecture (ASA) for robotics applications, which extends the original simplex architecture from the domain of reliability engineering by enabling the use of machine learning-based solutions to common robotics problems, like path and trajectory planning with and without obstacle avoidance. To demonstrate the applicability of the ASA, a novel analytic path planning method based on the Lamé curve equation was introduced and evaluated experimentally in a simulation environment and with a real 6-DOF robot. The results of this evaluation suggest that the proposed approach is suitable for human–robot collaboration scenarios in manufacturing, where strict safety norms and standards usually reduce the flexibility of interactions between humans and robots.

Compared to other variants of the simplex architecture, which builds on reinforcement learning, such as the neural simplex architecture (NSA), the ASA builds on supervised learning. This ensures higher levels of reliability and safety during runtime, because the ML model used as the high-performance planner can be trained using artificially generated data generated by verified algorithms. During operation, additional training data is generated and labeled using the outputs of a reliable acceptance test.

The proposed approach is simple to implement. This concerns all elements of the system, from the architecture to the ML model training procedure and the geometric path planning algorithm. This makes the approach robust and practical in a variety of human–robot collaboration scenarios. The source code implementing the proposed approach and that of the tools used in the evaluation is available online for free under the Apache 2.0 license (see Data Availability Statement).

## Figures and Tables

**Table 1:** Evaluation results—running time, path length, and smoothness.

Measurement	ASA (*f_ML_* + *f_opt_*)	ASA (*f_ML_* + *f_fast_*)	*f_opt_*	*f_fast_*
*t_p_* (ms)	1621	33	37292	786
*t_w_* (ms)	38.5	42.5	178.5	108.5
*t_m_* (s)	5.78	5.20	45.5	6.97
*p_f_*	1.28	1.31	1.23	1.34
*s_f_*	0.41	0.43	0.49	0.56

**Table 2:** Comparative performances of the proposed method with respect to those of four other motion planning algorithms [26,27,31,57]. SAC: Soft Actor-Critic, DOF; degrees of freedom, ASA: adaptive simplex architecture, and MPC: model predictive control.

Planning Method	DOF	Dynamic Obstacle	(Re)Planning Time	Success Rate	Path Overhead	Neurons	Complexity
ASA (*f_ML_* + *f_fast_*)	6	Yes	0.78 (ms)	100% *	Low	118	Low
MPC	6	Yes	7.4–41.1 (ms)	100% *	Low	−	High
SAC-3 + 3	3 + 3 (dual)	No	138.5 (ms)	100%	Low	7254	High
SAC-7 + 7	7 + 7 (dual)	No	−	89.7–92.9%	Low	5815	High
RRT Connect	6	No	144–200 (ms)	100%	High	−	High

* In some situations in which an imminent potential collision is detected, the respective planner achieves the stated reliability level by waiting until the obstacle moves away.

## Data Availability

The source code of the proposed ASA is part of the Assembly tool, which is available online: https://github.com/CoMeMak/assembly (accessed on 21 March 2021).
